# Acquisition of turn-taking in sign language conversations: An overview of language modality and turn structure

**DOI:** 10.3389/fpsyg.2022.935342

**Published:** 2022-08-08

**Authors:** Laura Horton, Jenny Singleton

**Affiliations:** Department of Linguistics, Stony Brook University, Stony Brook, NY, United States

**Keywords:** turn-taking, language modality, pragmatics, language acquisition, sign languages

## Abstract

The task of transitioning from one interlocutor to another in conversation – taking turns – is a complex social process, but typically transpires rapidly and without incident in conversations between adults. Cross-linguistic similarities in turn timing and turn structure have led researchers to suggest that it is a core antecedent to human language and a primary driver of an innate “interaction engine.” This review focuses on studies that have tested the extent of turn timing and turn structure patterns in two areas: across language modalities and in early language development. Taken together, these two lines of research offer predictions about the development of turn-taking for children who are deaf or hard of hearing (DHH) acquiring sign languages. We introduce considerations unique to signed language development – namely the heterogenous ecologies in which signed language acquisition occurs, suggesting that more work is needed to account for the diverse circumstances of language acquisition for DHH children. We discuss differences between early sign language acquisition at home compared to later sign language acquisition at school in classroom settings, particularly in countries with national sign languages. We also compare acquisition in these settings to communities without a national sign language where DHH children acquire local sign languages. In particular, we encourage more documentation of naturalistic conversations between DHH children who sign and their caregivers, teachers, and peers. Further, we suggest that future studies should consider: visual/manual cues to turn-taking and whether they are the same or different for child or adult learners; the protracted time-course of turn-taking development in childhood, in spite of the presence of turn-taking abilities early in development; and the unique demands of language development in multi-party conversations that happen in settings like classrooms for older children versus language development at home in dyadic interactions.

## Introduction

The task of transitioning from one interlocutor to another in conversation – taking turns – is a complex social process. Interlocutors who do not have the floor must process and comprehend ongoing turns, accurately anticipate when the person occupying the floor will provide an opportunity or solicit an opening for a turn shift, and, simultaneously, plan their own contribution (De Ruiter et al., 2006; Levinson, 2016). Following a turn change, interlocutors are expected to provide a turn that is both temporally and semantically contingent; language users assume that turns will change rapidly and that sequential turns will be related to prior utterances.

In spite of the social and cognitive demands, turn-taking in adult conversations often proceeds smoothly and with considerable efficiency (Sacks et al., 1974; Stivers et al., 2009; Levinson and Torreira, 2015). And where breakdowns in interaction occur, they are typically remedied or repaired rapidly (Dingemanse et al., 2015). The presence of turn-taking abilities early in both ontogeny and phylogeny, as well as the cross-linguistic consistency of turn-taking patterns and timing, has led some researchers to suggest that it is a core antecedent to human language and a primary driver of an innate “interaction engine” that underlies cross-linguistic similarities in some aspects of conversational exchange (Levinson, 2006, 2019).

In this article, we consider evidence that this ‘‘universal’’ human skill extends across linguistic modalities, from oral/aural spoken languages to visual/manual signed languages, as well as evidence for the presence of turn-taking abilities early in infancy for hearing children acquiring spoken languages. Based on the literature on turn-taking across modalities and in development, we discuss predictions for the development of turn-taking for deaf^1^ and hard of hearing (DHH) children acquiring signed languages. Prior work offers suggestions about the implications of language modality and development for turn-taking, but we also introduce considerations unique to signed language development – namely the heterogenous ecologies in which signed language acquisition occurs for DHH children.

The communicative ecologies for language acquisition that DHH children encounter vary across numerous characteristics but we focus on variation in two aspects: setting/interlocutors and language type. In terms of the settings for sign language acquisition, we discuss the difference between language acquisition at home with family members versus language acquisition at school with teachers and peers. In terms of language type, we discuss sign languages that vary along the following dimensions: the age of the language, the size of the community of users, the existence and availability of deaf education, access to medical technologies like hearing aids and cochlear implants, as well as prevailing ideologies about ‘‘best practices’’ for language development of DHH children. Although a variety of terms have been proposed to categorize sign languages,^2^ for this article we will use the term *local sign languages*, often from smaller communities of signers with a shorter history of use, that are used primarily in the home or informal settings, in contrast with *national sign languages*, often used by larger, geographically dispersed communities of signers with a longer history of use both at home and in institutional settings like schools. We review the acquisition of turn-taking for DHH children in three settings – two settings from communities with a national sign language and one setting from communities where there is no national sign language in use. In communities with a national sign language, we discuss: family socialization of national sign languages at home and classroom socialization of national sign languages at school. In communities without a national sign language, we discuss family socialization of local sign languages at home. We suggest that there are unique challenges for each of these groups of DHH children, based on their differential access to the language in their environment with a particular focus on three factors that could significantly impact the trajectory of turn-taking development in DHH children learning a sign language. These factors include: (1) language modality—acquiring a visual/manual language, (2) ontogeny—development as a child learner, and (3) socio-cultural factors—characteristics of the acquisition ecology. We suggest that more work is needed to account for the diverse circumstances of language acquisition for DHH children and to consider the role of both modality and unique socialization contexts for the learning of turn-taking in conversation. In particular, we encourage researchers to consider: visual/manual cues to turn-taking and whether they are the same or different for child or adult learners; the protracted time-course of turn-taking development in childhood, in spite of the presence of turn-taking abilities early in development; and the unique demands of language development in multi-party conversations that happen in settings like classrooms for older children versus language development at home in dyadic interactions.

We begin with an overview of studies of turn-taking structures in signed language conversations between adults (see section “Documenting Turn-Taking Structures in Sign Languages”) then discuss studies that have explored modality effects on turn-taking timing and cues by comparing spoken and signed languages directly using either experimental or naturalistic adult conversational data (sections “Language Modality and Turn-Taking: Turn Timing” and “Language Modality and Turn-Taking: Cues to Turn Changes”). We then turn to studies of turn-taking in acquisition, providing a brief overview of work on turn-taking development in spoken language acquisition (section “Acquiring Turn-Taking Structures: Spoken Language Development”). In the final section we discuss sign language acquisition in the three social ecologies introduced above: (1) acquisition of a national sign language at home; (2) acquisition of a national sign language at school; and (3) acquisition of a local sign language at home. While there have been few studies dedicated to turn-taking in sign language acquisition, we review studies of interactional skills necessary for turn-taking like attention-getting and we discuss areas where future work could provide important insights for turn-taking development in the visual-manual modality.

## Documenting turn-taking structures in sign languages

While many researchers of social behavior (along with travelers and language users who have encountered other dialects and languages) have expressed intuitions that turn-taking patterns vary widely across cultures and languages, the broader paradigm of alternating turns between two or more interlocutors in many tasks, conversation or otherwise, seems to be a human universal (Levinson, 2006, 2019). The tension between “innate” universal principles that guide all interaction and local standards for conversational exchange is evident in much of the work on turn-taking, particularly in the substantial body of work on turn-taking in spoken languages. Early work focused on both the behavioral cues (Yngve, 1970; Duncan, 1972; Duncan and Fiske, 1977) associated with turn shifts, as well as the broader principles (Sacks et al., 1974) governing turn alternations. Recent studies have explored the psycholinguistic mechanisms underlying turn-taking practices (Garrod and Pickering, 2015; Levinson and Torreira, 2015) as well as the multimodal aspects of turn-taking (Mondada, 2019). Much of this work suggests that the general principles of turn-taking should apply broadly to all language encounters. As such, these general principles should extend across modalities to sign languages. Studies of turn-taking in signed language conversations have attempted to evaluate the compatibility between patterns observed in signed interactions and those described for spoken languages. Multiple studies that have documented the cues associated with turn-taking in naturalistic sign language conversations between adults. As mentioned above, children will have to notice and acquire these cues in development. Several of these studies are summarized in Table 1.

**TABLE 1:** Studies of turn-taking in national sign languages.

Study	Language(s)	*N* participants	Data source	Turn-taking behavior(s)
Baker, 1977	American Sign Language (ASL)	4	2 conversations (dyads)	Descriptive (transcripts)
Cibulka, 2016	Swedish Sign Language (SSL)	42	Free dyadic conversations, 20 sessions (1 h 50 min)	70 instances of sign suspension^1^
Coates and Sutton-Spence, 2001	British Sign Language (BSL)	8	2 conversations (multiparty)	Descriptive (transcripts)
de Vos et al., 2015	Sign Language of the Netherlands (NGT)	16	6 dyadic conversations 1 triadic conversation (11 h)	190 questions 104 polar questions 86 content questions
Girard-Groeber, 2015	Swiss German Sign Language (DSGS)	4	1 multi-party conversation (33 min)	382 overlaps, (reduced to 331 based on eye contact)
Groeber and Pochon-Berger, 2014	Swiss German Sign Language (DSGS)	3	1 multi-party conversation (90 min)	84 turn-final holds produced by one of three students
Manrique and Enfield, 2015	Argentine Sign Language (LSA) Corpus	23	Informal dyadic, multi-party conversations (1 h 50 min)	23 instances of “freeze look”^2^ (original dataset: 213 instances of Other-Initiated-Repair (OIR)
McCleary and de Arantes Leite, 2013	Brazilian Sign Language (Libras)	2	1 conversation (dyad) (3 min)	4 examples of overlap or near overlap
McIlvenny, 1995	Finnish Sign Language (FiSL)	Not reported	Dyadic, multi-party conversations	Descriptive (transcripts)

^1^Cibulka (2016) uses the term “sign suspension” to describe “moments in signed interaction when sign production is temporarily suspended” (p. 448). He notes that suspensions happen for a variety of reasons (overlap in turns, forgetting a sign, etc.) and documents the ways that they are resolved in interaction.

^2^“Freeze look” is the term that Manrique and Enfield use for a behavior observed in signed conversations when a signer has been asked a direct question and “holds still while looking directly at the questioner” (3). They argue that this a strategy for other-initiated repair in conversation, and prompts the signer to repeat their original question.

Due to the time-intensive nature of collecting and annotating sign language conversation data, studies often involve a limited number of participants and use fewer than five conversations as their dataset, many use a single dyadic or multiparty conversation (see Table 1). These studies consist of descriptive analyses (Baker, 1977; McIlvenny, 1995; Coates and Sutton-Spence, 2001) as well as quantitative studies of specific turn-taking phenomenon like overlaps (McCleary and de Arantes Leite, 2013; Girard-Groeber, 2015), polar questions (de Vos et al., 2015); and sign holds (Groeber and Pochon-Berger, 2014; Cibulka, 2016). These studies and their findings are discussed in greater detail below.

In a descriptive study based on data from two conversations in American Sign Language (ASL), Baker (1977) identifies a set of prosodic cues and practices that characterize initiating a turn, continuing or maintaining a turn, and signaling a shift in turn. These cues and practices vary based on whether the signer is the producer or addressee and are summarize in Table 2.

**TABLE 2:** Turn cues from American Sign Language (ASL) identified in Baker (1977).

		Sign producer	Sign recipient
Signers’ hands	Initiate turn	Raise hands out of rest position	Maintain own inactivity
	Continue/maintain turn	Not returning to rest position	Backchanneling (head nodding, smiling, postural shift, facial activity suggesting surprise, agreement, uncertainty, and lack of understanding)
	Shift in turn	Return to rest position	• Move out of rest position • Wave, index to producer, touching, initiating first turn, repeating first few signs until producer has yielded floor or suppressed turn-claim
Signers’ gaze	Initiate turn	(−)GAZE if statement (+)GAZE if question	(+)GAZE
	Continue/maintain turn	(−)GAZE	(+)GAZE
	Shift in turn	(+)GAZE (if not already (+)GAZE)	Switch to (−)GAZE, when speaker is (+)GAZE
Optional cues	Initiate turn	• Wave to addressee • Index to addressee • Head/postural lean forward	
	Continue/maintain turn	• Increase in signing speed • Fill pause with movement • Hold last sign	• Index (point) to producer • Short repetitions of some of the producer’s signs
	Shift in turn	• Decrease signing speed near end of turn Call for response: • Palm up toward addressee • Indexing addressee (end of turn) • Holding last sign (questions) • Raising last sign (questions) Question intonation (face or body)	• Increase in size/quantity of backchanneling • Palm orientation change

The prosodic cues that Baker identifies primarily relate to the position of the signers’ hands, their eye gaze, and their signing size and speed.^3^ She describes three possible rest positions for signers’ hands, including full-rest, half-rest, and quarter-rest (Baker, 1977, p. 219), noting that a signer often signals their intention to interrupt or initiate a turn by altering the position of their hands (from full-rest or half-rest) and by changing their palm orientation. Significantly, many of the cues that Baker identifies have been excluded in subsequent studies of sign language conversations. Many studies exclude “preparatory” movements of the hands and arms when attempting to measure turn timing, for example. Thus, it remains unclear whether these cues are significant for signers, regardless of their age.

Many of the cues from Baker serve different functions depending on who produces them – *producer* or *addressee*. For example, if the *addressee* makes and/or maintains eye contact with the producer (+)Gaze, this suggests that they are ready for the producer to initiate or continue a turn. If the *producer*, however, makes eye contact with the addressee (+)Gaze, it often means that they are about to yield their turn. For child signers, this means they must acquire a complex set of signals that are contingent on their current status in an interaction. If a child signer is an addressee, making eye contact with their interlocutor (+)Gaze, will indicate something different than if they were currently the active signer.

Later studies have contested some of the claims in Baker. In their study of British Sign Language (BSL) conversations, for example, Coates and Sutton-Spence suggest that prior work on conversation structure, particularly turns, focused too much on conversations between dyads and conversations in formal settings like classrooms (p. 526). This led sign language researchers (e.g., Baker, 1977; Mather, 1996) to assert that signers obligatorily establish eye contact with their interlocutor(s) prior to initiating a signed turn. Coates and Sutton-Spence suggest that this may have been stated too strongly, and could be rephrased, “By ‘cannot’ [start a turn without eye contact] they clearly mean that optimum communication will not occur without the elaborate attention-getting they describe… we must understand Baker’s and Mather’s use of ‘cannot’ to mean ‘it would not normally make communicative sense for a signer to initiate a turn without eye contact with the addressee”’ (513).

In a study focused on the sequential context of turn overlaps in Swiss German Sign Language (DSGS), Girard-Groeber (2015) explored whether overlaps in sign language conversations occurred in “orderly” or predictable places in turns, as suggested for spoken language conversations (Jefferson, 1984, 1986). In particular, they asked whether sign turn overlaps tend to happen in the middle of *turn construction units* (TCUs) or if they were more common at *turn relevance places* (TRPs) and possible points of completion. In the DSGS conversation that Girard-Groeber analyzed, signers overlapped most frequently at TRPs and possible points of completion (79.4% of all overlaps).

Girard-Groeber found substantial overlap of signed turns, even though only turns that overlapped the stroke phase of signs were included in the analysis. If a signer raised their hands to prepare to sign as another signer occupied the floor, this was excluded from the analysis because it was considered the preparatory phase of the sign. This finding contradicts some earlier claims about the significance of the current signer terminating a turn by returning their hands to a full “rest position.” Girard-Groeber (2015) noted that signers often did not wait for the current signer to fully relax their hands, “Rather they fine-tune their turn-beginnings to the end of grammatical and prosodic units” (p. 205), a pattern noted for spoken languages as well (Selting, 1996). Based on Girard-Groeber’s claim, child signers may not be able to use hand position as a reliable cue to turn transitions (if they are expected to begin turns before their interlocutor has lowered their hands fully to a rest position). In spite of the finding that signers frequently overlapped their turns, Girard-Groeber suggests that these overlaps are “orderly,” happening at predictable moments in the conversation, and that signers are, therefore, still orienting to the “minimal overlap, minimal gap” principle discussed in the introduction.

McCleary and de Arantes Leite (2013) also argue that signers of Brazilian Sign Language (Libras) are motivated by the one-at-a-time or “minimal gap, minimal overlap” principle, based on an analysis of four examples from a conversation between two friends. They identify several “overlap resolution devices” (ORD’s), including: emphatic articulation of a sign to attract the attention of an interlocutor (p. 140), slowed signing speed (when one signer notices that his conversation partner has initiated a shrug and palm-up gesture) (p. 135), abruptly cutting off a sign (p. 135), explicitly asking for a partners attention with a wave or sign (p. 144) and lastly, lexically marking the start of a long turn with particular lexical items, like the sign “example” (p. 144).

Studies of naturalistic signed language conversations between adults generally suggest that there are specific signals associated with turn transitions as well as strategies for resolving overlapping turns when they occur, and while there is some evidence that turn overlaps happen frequently in signed conversations, it seems that these overlaps happen in similar places in conversational turns to spoken languages. We now turn to corpus and experimental studies that have compared spoken and signed language data directly to explore modality effects for two aspects of turn-taking: turn timing and cues to turn changes.

## Language modality and turn-taking: Turn timing

The study of turn-timing affords researchers a way to quantify and compare across disparate languages and social settings; but researchers face a challenge when determining what unit should be measured and compared. Early studies pointed out a basic principle of turn-taking—turn-length is rarely pre-determined in conversations. Regardless of the length of the preceding utterance, however, the transition between interlocutors happens across conversations and circumstances. In terms of timing, this transition could occur in one of three ways, (1) interlocutors could transition turns seamlessly, with no gap; (2) interlocutors could transition turns but there could be a gap with no speaking or signing; (3) interlocutors could overlap their turns, for a number of reasons, including (but not limited to) confusion about who will next occupy the floor, failure to end a turn when expected, or interruptions. Sacks et al. (1974) suggested that, in general, turn transitions are guided by a *one-at-a-time principle* such that language users use strategies to minimize gaps and overlaps between turns.

### Turn timing in spoken language conversations

Stivers et al. (2009) tested the *one-at-a-time principle* in a comparative study of ten languages that varied in their linguistic type (word order, sound structure, and grammar) as well as contexts of use (social structure). Using video recordings of naturalistic conversations, they measured the “response offset” – the temporal relationship between turns – in polar question-response sequences.

In their sample, response offsets were brief – for all languages in the dataset the mean was +208 ms – however there was a continuum of faster versus slower average response offsets across the sample (Japanese speakers had the fastest mean time for turn transitions at 7.29 ms while Danish speakers had the slowest at 468.88 ms). They found that four factors, including: answering, response type, non-verbal behaviors, and the presence of speaker gaze were significant predictors of response offset variation independent of the language spoken. Confirmation responses were faster than disconfirmation, responses with non-verbal behaviors (head nods, shakes, and squints) were faster than vocal-only responses, and responses were faster when the questioner gaze was directed to the addressee. Overall, Stivers et al. (2009) concluded that their data support a “universal system hypothesis,” (p. 10,589) and that users of all the languages they surveyed attempted to minimize both overlaps of turns and gaps between turns as predicted by Sacks et al. (1974).

Even though response offsets may be quantitatively similar across diverse communities of language users, humans appear to be remarkably attuned to the timing patterns in the language they use most often. Thus, within the overarching principle of “minimal gap, minimal overlap” (Schegloff, 2016), even small shifts in response offsets are perceived as significant divergences for language users from outside that community, “speakers of all languages aim at minimizing significant delays relative to the specific rhythm of that language in conversation… what constitutes a subjectively notable delay involves greater absolute duration in some languages than in others” (Stivers et al., 2009, p. 10590). Further, it remains to be established whether the patterns observed in the Stivers, et al. study extend to other utterance types (recall that they limited their dataset to polar question-response sequences) and across language modalities.

### Turn timing in sign language conversation

In a subsequent study, de Vos et al. (2015) explored whether Stivers et al.’s claim of a “universal system hypothesis” for turn-taking extended cross-modally. The researchers conducted a quantitative analysis of turn-timing in Sign Language of the Netherlands (NGT). As in Stivers et al. (2009) the sample was limited to polar question-answer pairs.

Similar to previous studies of sign language turn-taking, researchers encountered challenges when measuring sign length and identifying sign boundaries. This challenge of what “counts” in measures of turn boundaries is not limited to signed languages. Although gestural cues and inbreaths have been considered potential cues to turn units in oral/aural languages, Schegloff (2000) excludes them, describing them as “preparation for speaking but not part of speaking” (p. 50). Researchers have suggested that “preparatory movements” or the articulators (hands and arms) in sign languages are analogous to inbreaths (McCleary and de Arantes Leite, 2013, p. 133), and have thus excluded them from their analyses.

In de Vos et al. (2015), researchers compared two measures of sign boundaries (see Table 1 for participant and study information). The first method for annotating signs, termed “sign-naïve boundaries,” accounts for all movement phases of a sign. Phases were annotated based on gestural coding system from Kendon (1972, 1980, 2004) and Kita et al. (1998) and included: preparation, stroke, hold, and post-utterance retraction. The second method for annotation signs, termed “stroke-to-stroke turn boundaries” measures a sign for only the “stroke” movement phase that is “lexically specified.” Researchers coded from the last frame at which the lexically specified handshape was formed for the sign, stating “the start of the initial stroke (the ‘content’ part of the manual gesture) as the turn beginning as it most directly reflects the phonological content of a sign” (de Vos et al., 2015, p. 2). de Vos et al. use these measures to explore whether there are significantly more overlaps in signed conversations compared to spoken conversations and whether turn overlaps last longer or have a similar duration to overlaps in spoken conversation turns.

The proportion of turns that overlap, based on “sign-naïve turn boundaries” in the NGT sample is 82.2%. This proportion is significantly higher than the proportion of overlapping turns in the cross-linguistic spoken languages sample from Stivers et al. (2009) (which ranged from 13.5% overlapping turns for spoken Lao to 40% overlapping turns for spoken Japanese). The proportion of turns that overlap based on “stroke-to-stroke” boundaries was 29.8%, which was not significantly different from the spoken languages sampled. Similar differences were found for turns that had a significant gap (more than 120 ms). See Table 3 for a summary of turn overlaps and gaps from the study.

**TABLE 3:** Turn overlaps and gaps in Sign Language of the Netherlands (NGT) versus spoken languages.

Language	Turns with significant overlap*	Turns with significant gap*
NGT (sign naïve)	0.82	0.58
NGT (stroke-to-stroke)	0.30	0.17
Spoken Japanese	0.40	0.41
Spoken Dutch	0.31	0.49
Spoken Lao	0.13	0.73
Spoken Danish	0.16	0.72

*“Significant” gap/overlap duration threshold from Heldner (2011), based on a sample of spoken Dutch, judged by native Dutch speakers.

Data for NGT are from: de Vos et al. (2015, pp. 7–8).

Data for spoken languages are from: Stivers et al. (2009) and Heldner (2011).

When de Vos et al. compared “sign-naïve” and “stroke-to-stroke” measures of turn timing directly, they found turn offsets based on “sign-naïve boundaries” had, on average, lengthy overlaps between turns (mean −812 ms, negative boundary measures reflect overlapping turns). When turn offsets were based on “stroke-to-stroke” turn boundaries, there were, on average, short gaps between turns (mean 307 ms, positive boundary measures reflect a gap between turns) (see Table 4 for comparison of turn timing with data from Stivers et al., 2009). This finding underscores the implications of decisions made for annotating signs. If signs are annotated one way (using “sign-naïve” boundaries), sign languages have a very different distribution from spoken languages, specifically when using this method, sign language conversations appear to have longer overlaps and more frequent overlaps than spoken language turns. If signs are annotated using a different method (using stroke-to-stroke boundaries), then the distribution of turn overlaps in sign language conversations looks similar to spoken language conversations – there are relatively few overlaps and they are short in duration. The challenge of when signs should be considered to start or stop (and as a result, overlap) thus has significant effects on the analysis of sign turns.

**TABLE 4:** Turn timing in Sign Language of the Netherlands (NGT) versus spoken languages.

Language	Mean turn transition time (ms)
NGT (sign naïve)	−812
NGT(stroke-to-stroke)	307
Spoken languages (all languages)	208
Spoken Japanese	7
Spoken Dutch	109
Spoken Lao	420
Spoken Danish	469

Data for NGT are from: de Vos et al. (2015, pp. 7–8).

Data for spoken languages are from: Stivers et al. (2009) and Heldner (2011).

The findings from the NGT sample of polar questions, both in terms of precise timing of turns and the resulting proportion of turns with significant overlaps or gaps (again, based on a threshold of 120 ms) led the authors to conclude that “…it is therefore plausible that preparatory and retraction movements in signed conversation are best seen as parallel to the pre-beginnings and post-completion elements of spoken turns (cf. Schegloff, 1987), and that TRPs [turn relevance places] are best approximated by the end of the last stroke” (de Vos et al., 2015, p. 9). Although this study of sign language turns offers a detailed, quantitative analysis of turn timing, it is one of the few studies that attempts to quantify sign turn timing. It would be useful, in future studies, to use a cross-linguistic sample, similar to that studied for spoken languages (Stivers et al., 2009) to assess whether, similar to spoken languages, sign languages have consistent turn timing cross-linguistically.

Quantitative studies of turn-taking in national sign languages highlight the challenges of directly comparing spoken and signed languages, particularly when using precise measures for timing. Analyzed one way, the NGT sample suggests that overlapping turns in sign language conversations are more frequent and last longer than spoken language overlaps. But analyzed differently, NGT looks very similar to spoken languages, both in its exact timing and in the proportion of turn changes with overlaps and gaps. For DHH children acquiring a sign language, the results are somewhat inconclusive about the relationship between language modality and turn-timing. If children are attentive to the “stroke-to-stroke” cues that de Vos et al., coded, then they will be acquiring a system with similar timing patterns to spoken languages. If, however, they use cues like the preparatory movements of signers’ hands and arms or gaze, then it could be argued that they are acquiring a system with considerably more overlapping of turns than spoken language exchanges.

## Language modality and turn-taking: Cues to turn changes

Studies of turn timing have explicit criteria for isolating the unit of analysis: the person currently holding the floor finishes a turn and shifts to a different signer or speaker, but it has been harder for researchers to isolate and measure the cues that language users are producing and perceiving to anticipate turn beginnings and ends. When there is an exchange of turns, it is possible to measure the offset – gap or overlap – between language users. This consistency in the unit of analysis enabled researchers to compare across languages directly, both in a large cross-linguistic sample, and across language modalities between signed and spoken languages.

Early work by Sacks et al. (1974), mentioned above, proposed that there were two units that existed –TCUs and TRPs, where turns exchanges were possible (but not necessary). The original description of these units was quite vague, “Unit-types for English include sentential, clausal, phrasal, and lexical constructions” (Sacks et al., 1974, p. 702) and despite the broad list of possible unit types, much of this early work emphasized syntactic units as the primary cue to turn completion. In a subsequent study, however, using naturalistic conversational data, Ford and Thompson (1996) showed that TCU in spoken language conversations depended on a combination of syntactic, intonational and pragmatic cues. Further studies explored other turn cues, including pauses (Maynard, 1989), and prosody (Local and Kelly, 1986; Couper-Kuhlen and Selting, 1996; Caspers, 2003). Additional cues that undoubtedly play a role in cueing turns in face to face interaction come from the co-speech gestures that speakers produce. Much of the early work in conversation analysis on turn structure used telephone calls, precluding co-speech gestures as a source of turn information (Sacks et al., 1974). However, there is also a substantial body of work on the role of non-verbal cues in interaction, including eye gaze and gestures (Kendon, 1967; Goodwin, 1981; Bolden, 2003; Rossano, 2013). Due to space constraints, here we focus primarily on linguistic cues to turn structure, but note that non-verbal cues for conversational turns may be particularly critical for DHH children in hearing/speaking families. In the next section, we review studies that have developed experimental paradigms to directly test the role of different kinds of cues for turn prediction.

### Turn cues in spoken language conversations

In studies assessing the role of different kinds of cues for conversational turns, researchers often manipulate language data to control the amount of prosodic and lexical information participants can access. For example, by flattening the intonational contour of a recorded spoken language conversation. Participants are then asked to press a button when they think the current speaker has finished their turn (De Ruiter et al., 2006). In their study using naturalistic conversations recorded in Dutch, De Ruiter et al. (2006), found that participants were able to accurately predict the end of speakers’ turns when listening to audio with flattened pitch. Participants were less accurate at predicting the ends of turns when they had access to the intonational contours but not the lexicosyntactic information in the conversations. These results led the authors to conclude that for adult speakers, lexicosyntactic information is necessary, and possibly sufficient, for predicting turn ends. Most studies corroborate this finding for the role of lexicosyntactic information, but it is difficult to disentangle prosody from syntax (Ford and Thompson, 1996). A later study that varied both syntax and intonational cues (Bögels and Torreira, 2015) found that participants used information from both lexicosyntactic boundaries and intonational phrases to determine when turn ends would happen, and that they frequently produced errors when intonational phrases suggested a turn end in the midst of multi-utterance turns. In a study of English-speaking adults and children (discussed more in section “Acquiring Turn-Taking Structures: Spoken Language Development” below), Casillas and Frank (2017) also found that participants used both lexicosyntactic and prosodic information as cues to turn changes. Experimental studies of turn-taking are difficult in part because of the effort required to generate naturalistic stimuli. As a first step, researchers must get recorded language data that approximates natural conversation, but can be recorded and subsequently edited to change the information available from lexical content or prosodic content. In the next section we discuss the first study that attempts to use a similar method for sign language data.

### Turn cues in sign language conversations

Due to the difficulty of constructing stimuli, as well as recruiting participants, there have been very few studies of turn prediction in sign languages. Here we review de Vos et al. (2022), one of the first studies that uses similar methods to the studies discussed above, in which participants viewed signed naturalistic dyadic conversations between adult signers of NGT and pressed a button when they thought the current signer was about to end their turn. The researchers compared signers and non-signers to determine (1) whether participants could accurately anticipate turn ends, (2) whether participants were more likely to anticipate turn ends that contained questions, and (3) whether signers were more likely to anticipate turn ends in questions that included non-iconic question markers (lexical items) from NGT. They found that all participants (early-exposed signers, late-exposed signers, and non-signers) were able to accurately predict the ends of turns in the clips from NGT conversations. All participants were also more accurate for trials that contained questions. However, only early-exposed signers were significantly better at anticipating turn ends marked with NGT question lexical items. The researchers suggest that their findings lend support for Levinson’s (2006) interaction engine hypothesis because even non-signers who did not have any experience with NGT were sensitive to communicative intent in signed conversations. There also seems to be a widespread sensitivity to questions, or “response-eliciting” cues, above and beyond language-specific elements like lexical items.

For DHH children acquiring a sign language, this finding suggests that learners will have access to some cues for turns, even without early exposure to a sign language. They will not, however, be able to access all of the necessary cues without a language model and linguistic input.^4^ In particular, non-iconic lexical items that are not based on gestural patterns in the speech community, will not be available to them. Thus far we have focused on the relationship between turn-taking and language modality, but turn-taking patterns also vary across development or acquisition. We provide a brief overview of the work on acquisition of turn-taking in spoken languages before we introduce work on turn-taking and related communicative skills in signed language acquisition.

## Acquiring turn-taking structures: Spoken language development

Beginning in the 1970s, a considerable body of work was developed focusing on the development of turn-taking in early childhood (Bates, 1976; Snow and Fergueson, 1977; Ervin-Tripp, 1979; Ochs and Schieffelin, 1979; Garvey and Berninger, 1981). Here we discuss studies that have focused on the same aspects of turn-taking reviewed for adult language users: turn timing and turn cues.

The onset of turn-taking in infancy and childhood was initially debated, some early work debated the agency of infant children, who were observed to exchange vocally with caregivers well before they were able to produce language (Snow, 1977). Researchers have since taken measurements based on audio recordings of these exchanges to show that infants appear to be agential, “responding” rapidly to their mothers’ vocalizations with timing that suggests their vocalizations are contingent on, or responding to, their mothers’ (Gratier et al., 2015; Hilbrink et al., 2015). In Table 5, we present some of the timing data that have been reported from longitudinal studies across development of children engaged in different configurations of dyadic conversation, including adult–child and child–child interactions.

**TABLE 5:** Timing data (gap length between turns) from infants and children in conversation.

	0;4	0;9	1;6	1;8–1;9	2;4–2;5	2;10–3;3	3;0–3;1	3;3–3;5
Mother–infant^1^	326–921	542–3,297	485–1,270					
Caregiver–child^2^				844–1,017 (867)	446–1,738 (686)		357–894 (571)	292–619 (523)
Child–child^3^						900–1,500		

^1^ Mother–infant data are from Hilbrink et al. (2015), range of median gap time for infants, measured in ms.

^2^ Caregiver–child data are from Casillas et al. (2016), shortest and longest mean gap for children, mean gap for all children in parentheses, measured in ms.

^3^Child–child data are from Garvey and Berninger (1981), median “switching pause” values in ms.

Recalling that on average adult speakers have a 200 ms gap between turns, it is clear that children are slower than adults in their early vocal exchanges. And while turn timing may not be entirely driven by adult communication partners, children do appear to be affected by their interlocutor, based on the gap times reported by Garvey and Berninger (1981) for conversations between 2- and 3-year-old child peers (900–1500 ms). The timing of gaps seems to be tightly connected to children’s developing communicative and linguistic competence – both Hilbrink et al. (2015) and Casillas et al. (2016) find that children slow down at critical points in development when they may be developing new communicative skills or engaging in more complex linguistic production.

Hilbrink et al. (2015) examined the gap duration between turns beginning at 3 months until the children were 18 months old. While the timing of mothers’ responses to their infants’ vocalizations remained relatively stable across the study, infant response time varied significantly across development. Infants initially responded quickly to their mothers’ vocalizations (range of 345–902 ms at 3 months), but they slowed down around nine months (542–3,297 ms). The authors attribute the increase in gap timing around nine months to developmental changes in infants “communicative and social understanding of interactions” (p. 255).

Based on a dataset of naturalistic conversations from 5 caregiver-child dyads between ages 1;8 and 3;5, Casillas et al. (2016) documented the gradual development of rapid turn-taking. The timing of turns was closely related to both the child’s age and the complexity of the turn. Children were able to reply more quickly to simple questions (yes/no) at younger ages and gradually developed the ability to respond to more complex questions across development. Casillas et al. suggest that increasingly complex questions from caregivers – and the increasingly complex answers they require – may entail more processing demands for the children. The authors note the dual contribution of comprehension –understanding the question– and production –formulating an answer – to processing demands on child speakers.

Studies of spoken language interactions with young children thus suggest that children do have the capacity to intentionally engage others from a very young age, prior to their ability to produce or comprehend language, lending support for the universal “interaction engine” (Levinson, 2019). However, this does not yield a straightforward ability to immediately engage in adult-like conversation. For DHH children acquiring a sign language, this work suggests that child signers may have precocious abilities to engage in alternating turns early in development, but also that they may not look exactly like adult signers in conversation until later in development. So far, no studies that we are aware of have attempted to measure turn timing in sign language conversations with children, a point we return to in the discussion.

In a study of children’s ability to anticipate turn changes, Casillas and Frank (2017) showed participants (both child and adult English speakers) videos of dyadic conversations between two speakers of one of five languages (English, German, Hebrew, Japanese, or Korean). The non-English conversations were used to provide participants with non-lexicosyntactic cues to turn boundaries (e.g., prosody, gesture, and phrase-final lengthening). Similar to studies comparing different cue types discussed in sections “Turn Cues in Spoken Language Conversations” and “Turn Cues in Sign Language Conversations” above, these stimuli were intended to test the role of lexicosyntactic and prosodic information as cues to turn exchanges, within the context of naturalistic conversation. Importantly, these stimuli also included gestural information since they were video conversations, unlike prior studies which involved listening to audio recorded conversations and pressing a button.

All participants in the study were affected by turn type (question versus non-question); they made more anticipatory gaze switches following questions. Children (child participants ranged in age from 3;0 to 5;11) were also affected by the language used in the video. Younger children made more anticipatory gaze switches while watching clips from English conversations than non-English conversations, suggesting that children need access to lexicosyntactic information to predict the ends of turns, but as they develop they get better at making use of non-lexicosyntactic information. Contrary to prior findings that children rely primarily on lexical or syntactic information to predict turn endings, this study suggests that children (and adults) have alternative strategies to predict the ends of conversational turns when lexical or syntactic information is unavailable to them.

In a follow-up study that more closely controlled the amount of prosodic and lexical information available to participants, Casillas and Frank (2017) found that young children (1;0–6;11) were spontaneously able to make turn predictions by age 2;0. Even at age 6;0, however, children were not as accurate at adults in their turn behavior predictions. The researchers conclude that children are aware of turn cues from a very young age, but develop an ability to make predictions based on these cues gradually across development. In particular, they emphasize that children seem to need access to lexical information, whether a turn contains a question or not, to achieve adult-like prediction behaviors and reiterate that it takes children several years to fully integrate all of the cues that contribute to effective turn taking monitoring and responsiveness.

Together with the results from studies of sign language turn prediction, studies of child speakers would suggest that DHH children should have some ability to anticipate turn changes in sign conversations from a young age. However, their ability to achieve adult-like efficiency in predicting turn changes will not occur until later in development. There is, further, a critical modality-based difference for DHH children acquiring a sign language versus hearing children acquiring a spoken language. While it is likely helpful for hearing children to be able to turn their head in time to see a speaker begin a turn in a spoken language conversation, DHH children will miss the linguistic signal completely if they do not direct their attention to the next signer in time to see the start of their turn. In other words, hearing children can hear a spoken language turn whether they are looking at the speaker or not, but a DHH children cannot see a signed turn if they are not looking at the signer. Whether gaze is a prerequisite of initiating a turn in adult signed conversations is somewhat contested, but for child signers this is a critical prerequisite for following and eventually entering into sign conversation. In order to follow signed conversation, child signers must recognize the cues and patterns of turns in signed turn exchanges. As noted in the introduction, researchers continue to debate whether the visual/manual modality of sign languages alters their turn-taking structure and whether this has implications for acquisition.

## Acquiring turn-taking structures: Signed language development

Spoken language acquisition happens with seemingly little effort on the part of caregivers and children. Hearing children are exposed to the language(s) spoken around them and gradually grow in their ability to comprehend and produce them. DHH children, however, are often in very different circumstances from hearing children. They are typically born into hearing families.^5^ where no one knows a sign language. They may be born in a community where there is not access to a national sign language or formal schooling for the deaf. In countries with universal hearing screenings at birth, children and their families are rapidly recruited into systems with support for medical interventions like hearing aids or cochlear implants and language intervention like speech therapy or sign language classes (Mauldin, 2016). And while there is a considerable body of work documenting spoken language acquisition for DHH children, both at home and at school,^6^ in this section, we focus on DHH children who are acquiring a sign language. We will explore turn-taking development for DHH children learning a national sign language at home from signing parents or grandparents; DHH children learning a national sign language at school from signing teachers and peers; and DHH children learning a local sign language at home.

### Deaf or hard of hearing children acquiring a national sign language at home

As discussed above, the majority of DHH children are born into hearing/speaking families (Mitchell and Karchmer, 2004). The small percentage of DHH children who are born to DHH signing parents offer insight into the language acquisition process when it occurs in the visual/manual modality with early and full access. In longitudinal studies of sign language development at home, researchers have observed that signed interactions between DHH parents and DHH children differ significantly from adult signed conversations. There have been several longitudinal studies of the sign language acquisition of DHH children at home with DHH signing parents, the studies cited in this section are summarized in Table 6 including the sign language used and the ages of the children observed. This is not intended to be an exhaustive list of all studies of sign language acquisition at home, but includes studies that specifically mention acquisition and development of turn-taking and attention-getting patterns in early signed interactions between DHH parents and DHH children.

**TABLE 6:** Studies of sign language development of DHH children of DHH parents.

Study	Language	*N* participants	Participant age(s)	Data
Harris et al., 1989	British Sign Language (BSL)	4 mother–child dyads, DHH mother and DHH child	Children observed at 7, 10, 16, and 20 months	Video recordings of free play (20 min)
Harris and Mohay, 1997	British Sign Language (BSL); Australian Sign Language (Auslan)	11 mother–child dyads; all DHH children; 5 DHH parents native users of BSL or Auslan; 6 hearing parents enrolled in Signed English program	18 months	Video recorded data of child and caregiver interacting at home or in a lab setting with toys (20–40 min)
Holzrichter, 2000	American Sign Language (ASL); Sign Language of Spain (LSE)	6 DHH children with DHH parents (3 from each language)	ASL children ages 2;5–3;10 LSE children ages 2;1–4;2	Video recordings of child playing with caregiver at home using toys, flashcards
Meadow-Orlans et al., 2004 data collected: 1988-89	American Sign Language (ASL)	20 DHH children with DHH parents Subset of 80 infant/caregiver dyads: 20 DHH children with Hearing parents 20 hearing children with DHH parents 20 hearing children with hearing parents	Children tested at 6, 9, 12, 15, and 18 months	video recordings of free play, still face/strange situation, interviews, developmental profiles
Pizer et al., 2011	American Sign Language (ASL)	3 DHH children with at least one native signing parent	9, 13, and 15 months (additional recordings at 17–18 months and 24 months for 2/3 participants)	10 min of video recordings of free play
Swisher, 1999	American Sign Language (ASL)	9 dyads DHH child with DHH caregiver Subset of Gallaudet longitudinal study (Waxman and Spencer, 1997; Meadow-Orlans et al., 2004)	Children observed at 9, 12, and 18 months	Video recordings of free play with toys

In their study comparing DHH and hearing infants from different contexts (hearing and deaf signing families; data collected at 6, 9, 12, 15, and 18 months), Meadow-Orlans et al. (2004) note.

*Clearly the pace of linguistic turn-taking in the first year of life is slower for dyads in which child and mother are deaf than for dyads in which both are hearing. This difference in pace is to be expected because deaf persons must divide their visual attention between exploring objects in the environment and receiving communications. This effect is not observed in adult conversations, but is a pervasive characteristic of signed conversations with infants and toddlers who have not yet developed the ability to make smooth changes in focus of visual attention (p. 162).*

Meadow-Orlans et al. suggest that DHH caregivers adjust the pace and timing of their turns to accommodate the visual attention of their DHH child. As discussed above, learning a sign language places different demands on the child in terms of visual attention. In contrast to spoken language input, which the hearing child can access with or without visual attention to the speaker, the child learning a sign language must see signing in order to perceive it, and they must be attentive to engage in turn-taking. Visual attention is thus a necessary prerequisite to turn-taking in sign language, and we include studies of visual attention management in this review. Studies of mother–child dyads suggest that deaf signing mothers make significant adjustments to their signing to engage their child’s visual attention. There are contradictory reports in the literature, however, regarding the strategies that DHH signing mothers use with their DHH signing children.

In many studies, researchers report that deaf signing mothers seem to adopt less overt strategies for capturing and directing their DHH children’s attention; this is reflected in both the amount of time that DHH mothers spent waiting for their child’s attention, as well as their use of explicit attention-getting signals. In a study of four mother-child dyads,^7^ Harris et al. (1989) found that mothers generally moved their signing so that it was within the child’s visual field, noting, “rather than manipulating the child’s focus of attention, the mothers tended to sign where the child was already looking” (p. 90). This pattern aligns with other studies of child-directed signing and a tendency to wait for the DHH child to look to the mother, rather than employ strategies to attract or redirect the child’s current focus of visual attention. Meadow-Orlans et al. (2004) characterize deaf signing mothers noting, “The picture of communication presented by Dd mothers was often one of watchful waiting and responding to their children’s interests when presented with an opportunity to communicate” (160). Observations about how deaf signing mothers choose to take turns in conversation with their young deaf children are supported by quantitative evidence that deaf mothers spent significantly more time waiting on their children (70% of a 3-min face-to-face dyadic exchange) when compared to hearing mothers of hearing infants (35%) and hearing mothers of infants with a hearing loss (16%) (Spencer et al., 1992, p. 72). These studies suggest that DHH signing mothers are often willing to spend a significant amount of time waiting for their DHH child’s visual attention, rather than actively seeking to change their child’s focus. This is reinforced by studies exploring the use of explicit attention-seeking signs and cues.

In a cross-linguistic comparative study of deaf children (ages 2;1–4;2) in deaf signing families from Spain and the United States, Holzrichter (2000) found that deaf parents^8^ used few attention-getting devices with their signing children, noting that “In general, parents of two-year-old’s seemed willing to wait for their children’s attention and to allow the child to set the pace of the conversation” (p. 66). Holzrichter compared 2-year-old and 4-year-old signers, reporting that all children were most likely to be engaged in mutual gaze with their parents for most turns (72–77% of turns across the sample), with parents looking away during turns with 4-year-old’s more often than 2-year-old’s. Holzrichter suggests that withholding or averting their gaze could be a strategy that parents of older children are using to maintain the floor, noting that the 4-year-old’s were much more active contributors to conversations, introducing new topics and actively competing for the floor (p. 64).

The results from Holzrichter are compatible with findings from an earlier study by Swisher (1999) that documented attention-getting strategies in 9 ASL-using infants who were recorded interacting with their mothers at 9, 12, and 18 months. Swisher found highly variable rates of attention-getting strategies from the mothers – some frequently tapped their children, waved toys at them, or moved their signing into the child’s line of vision, while others rarely engaged in these practices (see also Meadow-Orlans et al., 2004, pp. 184–186 for additional discussion of these results). Across the sample, however, children consistently became more responsive to these techniques. This was especially true for taps for attention, which were the most frequent strategy when the child was within reach of the mother. Average responsiveness to attention-getting strategies increased from 23 to 50 to 78% at 9, 12, and 18 months respectively (p. 34). Swisher notes that by 18 months, “Turn taking appeared to be more rapid, with children more often responding quickly and crisply to taps as well as shifting gaze frequently to their mothers” (p. 35).

In general, these studies suggest that deaf signing parents may be less focused on directing or attracting their child’s attention, and more attentive to where the child is already looking and adjusting their own signing, when necessary, to place it within the child’s field of vision or to comment on the target of the child’s visual attention. For the acquisition of turn-taking and early turn-taking patterns between DHH signing adults and young DHH children, this indicates that turn-taking may be quite slow and characterized by sustained breaks in interaction while the adult waits on the child’s attention before initiating the next turn. Deaf signing mothers may seek to provide targeted input and, in particular, are very careful to make sure that they have the child’s attention before they sign, a finding reported across numerous studies of signing conversations with young deaf children. Meadow-Orlans et al. (2004) note that deaf mothers (in Dd dyads) were “highly consistent in providing linguistic information when children responded to an attention signal by looking at the mother” and that mothers’ utterances were “highly responsive to their children’s visual attention focus (or the focus just before they looked up at the mother)…” (p. 160). When compared directly to hearing-hearing mother-infant dyads, some studies have found that deaf-deaf signing mother-infant dyads are characterized by quantitatively less input (Harris et al., 1989, p. 93; Spencer and Lederberg, 1997, pp. 224–225; Meadow-Orlans et al., 2004). However, most of these studies also report that the deaf signing infants achieve similar linguistic milestones at similar ages to their hearing peers.

Reports of patient, watchful waiting from deaf signing caregivers contrast somewhat with studies that report on more explicit or overt efforts to get the attention of deaf signing children or to elicit signing from them. Pizer et al. (2011), for example, report frequent use of sign repetition and sign lengthening in deaf signing parent-child dyads (children observed at 9, 13, and 15 months). They suggest that this is a strategy intended to prompt or elicit a response from the child signer. Similarly, in a comparison of DHH and hearing parents of DHH children (18 months), Harris and Mohay (1997) reported that only mothers who were DHH regularly attempted to elicit their children’s attention. As a result, these mothers had more frequent successful attention switches as well as failed attempts (p. 100–101).

Deaf or hard of hearing parents may vary considerably in their use of explicit strategies to manage and direct the visual attention of their signing children. This is likely also closely related to the social, physical, and cognitive development of the child. As Harris et al. (1989) point out, significant physical developmental milestones alter a child’s mobility and ability to change their own focus of attention. Many longitudinal studies document the ways in which signing parents change their strategies in response to their child’s changing capacities. This is reminiscent of the developmental milestones noted in Hilbrink et al. (2015) and Casillas et al. (2016), discussed in section “Acquiring turn-taking structures: Spoken language development”. In these studies of hearing children acquiring spoken languages, researchers suggest that changes in turn timing may be tightly linked to changing social abilities. In these examples, turn timing and responsiveness slowed down for children as they reached various cognitive and social milestones. While DHH children who acquire a national sign language at home will proceed through the language acquisition process along a similar timeline to spoken language acquisition (Newport and Meier, 1985; Lillo-Martin and Henner, 2021), and with similar parallel cognitive and social developmental milestones, DHH children who acquire a sign language at school^9^ enter this ecology at a much later stage of cognitive and social development, in addition to the differences between home and school social settings (Singleton and Morgan, 2006).

### Deaf or hard of hearing children acquiring a national sign language at school

We begin this section with a short vignette from a third-grade classroom at a state residential school for the deaf in the United States. Drawing on classroom observations from a longitudinal study, Ramsey and Padden (1998) provide several illustrative interactions between one student, Danny, who was a “newcomer” to this third-grade classroom, and his peers and teachers. At 9 years old, Danny was starting his second year at the school and the researchers observed that he had limited ASL proficiency and English literacy skills. They note that Danny was not only challenged by gaps in his ASL vocabulary and grammar, “Rather, Danny’s apparent inattentiveness and his difficulties with writing also involved his inability to follow signed discourse in a classroom setting” (p. 16). They provide a more detailed example of the kinds of challenges Danny faced that relate explicitly to turn-taking in the classroom,

Connie (the teacher) directed the class’s attention to a section on the worksheet listing the materials needed for the experiment. She opened the discussion with her WH-question pattern, signing “now,” pointing to the appropriate section on her overhead, and asking what it said… Danny and a number of other students raised their hands. Before anyone was called on, however, Danny dropped his hand and began fingerspelling “materials” to himself. He looked down at the worksheet to confirm the spelling, and continued fingerspelling to himself as Connie pointed to another student, Larry, in the back of the room. As a result, Danny missed Connie’s allocation of the turn to Larry, and when he looked around the room, could not locate him in time to see the answer (pp. 16–17).

The authors note that Danny loses track of the conversation, causing him to miss other students’ turns as well as the teachers’ instructions. Danny’s missing skills in discourse were particularly noteworthy to the authors because of his advanced age, but his difficulties closely resemble many patterns observed for younger DHH children in signing preschool classrooms who come from hearing/speaking families.

There have been several studies of children who are acquiring a national sign language in classroom settings. As mentioned above, the majority of DHH students are not receiving consistent sign language input at home and thus depend on the language input that they are exposed to at school to acquire the national sign language. Many studies compare students who do receive sign language input at home (deaf of deaf, DD, DoD) to students who are from hearing families (deaf of hearing, DH, DoH). In the following sections, we discuss these studies, summarized in Table 7.

**TABLE 7:** Studies of taking turns and getting attention in signing classrooms.

Study	Language(s)	N participants	Setting	Data
DeLuzio and Girolametto, 2006	American Sign Language (ASL)	4 children (3;3–4;7) (2 DHH, 1 CODA, 1 hearing child with deaf grandparents) 1 deaf teacher	Bilingual/bicultural preschool classroom (Toronto)	Video recordings (30 min total: 15 min. dramatic play, 15 min. playdough) -Type of attention strategy used by teacher -Intent of attention strategy -Child response
Graham and Tobin, 2020	American Sign Language (ASL), French Sign Language (LSF), Japanese Sign Language (JSL)		Signing kindergarten classrooms in the United States, Japan, and France	Video ethnography Discussions of ideologies of sign language with deaf teachers
Lieberman, 2015	American Sign Language (ASL)	7 children (1;9–3;3) (all deaf of deaf native signers) 5 adults (2 assistants, both deaf; 3 hearing, signing)	Signing preschool classroom in residential school for the deaf (1)	Video recordings of free play activities (30 h over three months) Strategies for getting attention (1,600 turns across all child participants; 477 peer initiations)
Mather, 1987	American Sign Language (ASL)	9 children in two classrooms (4 children deaf of deaf native signers) 2 teachers, 1 deaf native signer and 1 hearing signer	Signing preschool classrooms (2)	Video recording of story time Annotated use of two types of eye gaze to manage turn taking
Ramsey and Padden, 1998	American Sign Language (ASL)	1 focal student, class of 12 DHH students 1 teacher (deaf native signer)	Third grade classroom, state residential school for the deaf	Video recordings (35 h total, 20 observation days)
Singleton and Crume, this issue	American Sign Language (ASL)	6 children (all DHH) (3 children deaf of deaf native signers) 1 teacher, 1 aide (deaf, fluent signers)	Signing preschool classrooms (2)	Video recording of classroom activities Attention actions and participant cues used by teachers
Singleton and Morgan, 2006	American Sign Language (ASL)	3 deaf teachers	Bilingual/bicultural preschool	Video recordings
Smith and Sutton-Spence, 2005	British Sign Language (BSL)	10 children (3–5 years old) (all DHH) 2 teachers (deaf adults, BSL signers)	Signing nursery school, children attend full or half days	Video recordings (12 sessions) during free play and lunch Attention-getting strategies by teachers and children

A diverse range of methodologies have been used to study classroom interactions, including longitudinal engagement with a single classroom (Ramsey and Padden, 1998; Lieberman, 2015), sampling from different activities and spaces in classrooms (Smith and Sutton-Spence, 2005; DeLuzio and Girolametto, 2006), and comparing different types of students or teachers across classrooms (Mather, 1987; Singleton and Crume, 2010). Researchers have also used combinations of video recorded data as well as interviews with deaf teachers to explore language ideologies operating in these classroom spaces (Singleton and Morgan, 2006; Graham and Tobin, 2020). These studies document the specific attentional strategies that teachers employ, the efficacy of these strategies, and their beliefs about student language development in the classroom.

While many DHH children are receiving their primary language input in the classroom, the classroom ecology is remarkably distinct from the home context described for DHH children learning a national sign language at home (Singleton and Morgan, 2006; Graham and Tobin, 2020). One signing adult teacher (and often one additional signing teaching assistant) is tasked with the management of three or more young children. DHH children are thus embedded in a social context in which there are many competing demands on their visual attention and in which the majority of their interactions will be multi-party and they must compete for the floor. They are learning to manage their own visual attention, switching between the teacher, visual materials, and other signing students (Mather and Clark, 2012). Additionally, children in classroom settings are physically, cognitively, and socially more developed than the DHH infant who first encounters national sign language at home from their parent. We discuss the implications of these factors further in section “Discussion.”

#### Signing teachers and deaf or hard of hearing children

Conversations in classrooms diverge significantly from other social settings. In a pattern first identified by Mehan (1979), teachers frequently employ a structure known as Initiation – Response – Evaluation (or Feedback), or IRE. In this structure, the teacher poses a question (the initiation) for which they typically already have the answer, and solicit an answer from a single student or multiple students (the response), the teacher then provides an evaluation or feedback assessing the correctness of the student response. This structure has been widely documented in spoken language classrooms, including those with DHH children (Wood et al., 1982), but we know less about turn-taking patterns in signing classrooms with DHH students. Studies have documented the efforts of signing teachers in these classrooms to establish and direct the visual attention of students who are entering into the classroom conversation. As mentioned above, visual attention is a prerequisite for perceiving and, ultimately, entering into signed conversation turns. A signer not currently holding the floor, have visual access to (be looking at) the current signer, and, in the case of multi-party conversations, anticipate a change of turns and the location of the next signer so that they can shift their gaze to see the next turn. In this section we review some of the studies that have documented classroom discourse in early signing classrooms, focusing on this skill of shifting visual attention during sign conversation.

In contrast to the studies of DHH signing parents discussed in section “Deaf or Hard of Hearing Children Acquiring a National Sign Language at Home” that report that caregivers often used a strategy of waiting for their child’s attention, many studies of classroom sign language socialization document explicit attention management strategies used by signing teachers. These strategies are numerous; in a study of a British nursery school, Smith and Sutton-Spence (2005) develop an inventory of 39 different strategies that teachers and children used to attract attention. These strategies often target students, like Danny, introduced above, who enter the signing classroom with less previous experience following and contributing to signed conversations.

In a study of a signing preschool classroom, Singleton and Crume (2010) found that a deaf teacher and her deaf teacher’s aide directed many linguistic prompts toward the DHH students that signaled where to look (LOOK-AT-ME, READY?); however, the teachers used noticeably more physical/tactile prompts (tapping) toward the deaf children of hearing parents (DoH) who were not always anticipating where to look in the conversation. DoH students were also on the receiving end of “delay prompts” from the teacher in response to their repeated interruptions or trying to participate when it was clearly not their turn. The findings in this study suggest that by age 5, DoD appear to have internalized turn-taking patterns of ASL insofar as needing only linguistic cues like READY? from the teacher to signal where to look and also show low rates of interrupting the teacher. By contrast, DoH students still needed scaffolding to support their looking behavior and conversational participation.

In a similar study of teacher attention strategies in a signing preschool classroom in Toronto, DeLuzio and Girolametto (2006) evaluated how a deaf signing teacher used different types of attention strategies (tactile, visual, visual using an ASL sign, and observing/waiting) and whether these were used for different intents (initiating a conversation, continuing a conversation, or controlling a child’s behavior). They also evaluated the outcome of these attention strategies, finding that the teacher was most likely to use either tactile (tapping) or visual (waving) strategies, particularly when trying to gain students’ attention to initiate a conversation. The teacher did not often make attempts to continue or regain students’ attention in ongoing conversation, suggesting that many interactions were brief. In terms of the success of the four types of attention strategies, waiting was significantly less successful than any of the remaining three strategies (tactile, visual, and visual using an ASL sign). This finding is somewhat counterintuitive, given the extensive literature (discussed above in section “Deaf or Hard of Hearing Children Acquiring a National Sign Language at Home”) on patterns of interaction and turn taking between deaf caregivers and deaf infants and children.

In addition to manual strategies for managing attention, some studies have documented non-manual techniques that teachers use to manage student attention. In a study of two signing classrooms, Mather (1987) compares the use of different gaze strategies during a shared storybook activity by a deaf and a hearing teacher. Mather notes significant differences in the quality of turn taking in the two classrooms. She attributes these differences to the use of two types of gaze that indicate whether a question or comment is being directed to an individual student (I-GAZE) or to the entire group (G-GAZE). Mather suggests that the hearing teacher lacked proficient control of the two types of gaze to regulate turn taking in her signing and this led to confusion and misunderstandings with her students (p. 19).

Whether teachers are using manual or non-manual cues like eye gaze, the visual and conversational demands on students in the signing classroom setting are high. Studies from Smith and Sutton-Spence (2005), Singleton and Crume (this issue), and DeLuzio and Girolametto (2006) suggest that teachers do a lot of work to manage students’ attention to classroom discourse and to scaffold students’ attention so that they can follow and enter into the classroom conversation. Mather raises the additional consideration that some teachers may lack the signing proficiency to provide this scaffolding.

Beyond the individual strategies and cues that teachers employ, other studies have highlighted the significant role of deaf signing teachers, to provide more naturalistic interactions for deaf signing students than might normally happen in a classroom setting. In their comparative study of deaf signing preschools in the United States, France, and Japan, Graham and Tobin (2020) argue that deaf teachers are essential agents in the socialization of deaf children, not only in the acquisition of sign languages, but also of Deaf cultural norms of “eye gaze, attention elicitation strategies, joint attention, facial expressions, and body language” (p. 147) or what they describe as “deaf ways of being” (p. 147). Similarly, Singleton and Morgan (2006) highlight the role of deaf teachers in signing classrooms, who can offer students explicit reflections on the experience of being deaf and how to interact effectively with hearing people (p. 359). In terms of turn-taking, deaf teachers may be more attuned to novice child signers’ needs and can make the social practices and expectations that underlie successful sign conversations more explicit for students (Graham and Tobin, 2020, pp. 152–154). As Graham and Tobin note, “Teachers who have all five senses may not understand what it is like to only have four senses and how those individuals with four senses compensate in terms of enhanced communication information” (p. 158).

#### Signing with deaf or hard of hearing child peers

While many studies of adult-child conversations (both sign and speech) note that adults often scaffold interactions for the child participant, sometimes peer conversations between children do not proceed as smoothly. As noted in section “Acquiring turn-taking structures: Spoken language development”, for example, turn gaps between child peers at ages 2–4 were significantly longer than adult turn gaps (Garvey and Berninger, 1981). In a study of deaf children of deaf parents, Lieberman (2015) reports that by 19 months of age native signing deaf children are aware that they need to establish eye gaze before beginning a turn. Children very rarely proceeded with a turn if they did not have the visual attention of their conversational partner, but child signers also frequently “gave up and either walked away or made no further attempts to get the addressee’s attention” (p. 862). In terms of the success or failure of initiations, children had a similar success rate in their initiations with peers (64% successful) as they did with their teachers (65% successful). Notably, this success rate between deaf conversation partners is much higher than that reported for deaf children interacting with hearing children (Messenheimer-Young and Kretschmer, 1994; Deluzio and Girolametto, 2011).

In the Lieberman (2015) study, children had various strategies for attracting and maintaining the visual attention of their peer interlocutor including taps, object use, signs, actions, and physical approach. Even though waves are a very common strategy in adult signing conversations, children rarely used them in peer interactions (p. 861). To manage turns, children were strategic in their use of different techniques. If they were initiating a turn they were more likely to use taps or waves, but if their conversational peer was already attending they tended to use signs or gestures to sustain attention. These results suggest that, even from a very young age (19 months), DHH children who receive early sign language input acquire important turn-taking skills – like waiting for the visual attention of their interlocutor – and strategies – like tapping or signing to attract and sustain attention. To our knowledge, no studies have explored turn timing in these contexts, but it would be interesting to know how often these turns overlapped, or whether the gaps between turns were slower compared to adult signers (as has was found for spoken language interactions between hearing children at the same ages).

In general, there are few studies exploring the impact of late language acquisition, or language deprivation (Hall, 2017; Hall et al., 2019) on the development of pragmatic skills in signing DHH children. A recent overview study suggests that DHH children acquiring *spoken* languages show significant delays in pragmatic skills (Paatsch and Toe, 2014; Paul et al., 2020), but less is known about DHH children acquiring sign languages. In their discussion of language deprivation, Koulidobrova and Chen-Pichler (2021) advocate for a reconsideration of the systems developed by DHH children who do not receive early sign language input. They suggest that researchers take seriously the systems that DHH children develop in the absence of full input, which they describe as the “initial system.” It would be worthwhile for studies of these “initial systems” to document turn-taking and other pragmatic skills in addition to lexical and syntactic patterns.

For other domains of linguistic development, it is clear that early ASL exposure (before 6 months) can lead to native-like results, even for DHH children who are in hearing families [see Caselli et al. (2021) on vocabulary acquisition and Henner et al., 2016 for syntax]. In contrast, delayed sign language exposure may contribute to a range of language disfluencies in sign language comprehension and production, including in syntax (Boudreault and Mayberry, 2006), morphology, and processing (Mayberry, 2010). The relationship between sign language input and experience and pragmatic skills should be explored in future studies, a point we return to in section “Discussion” below.

### Deaf or hard of hearing children acquiring a local sign language at home

Deaf or hard of hearing children born into hearing families in countries with a national sign language enter communities with specific beliefs about appropriate and necessary interventions. In other countries the national sign language may not be as widely used, medical interventions may be less common, affordable, or accessible, and schools for the deaf may be geographically or financially inaccessible to DHH children. Without early hearing screenings, many families may not know that their child is deaf until much later, sometimes 6 or 7 years old. In this context, DHH children and adults often develop and use local sign languages to communicate with hearing relatives and friends. As mentioned in the introduction, there is immense variation in these systems, in terms of how many signers they have, the geographic spread of their use, and hearing people’s attitudes toward deaf people and signing. In this section, we consider implications for development of turn taking in sign language conversations for children in these settings. While extensive work has documented the lexical, morphological, and syntactic properties of many of these languages, fewer studies have focused on pragmatic practices like turn taking. We discuss studies that have described turn-taking in local sign languages used in Central and South America, as well as areas for future study.

Haviland (2020) provides a close analysis of several conversations between three deaf adult siblings in Chiapas, Mexico. In his description of “Z sign,” Haviland highlights the significant role of eye gaze in these exchanges, noting the ways that gaze direction is mobilized for referential and indexical purposes, as well as selection of the next participant in the conversation. Gaze can be used to designate the next signer, or to establish someone as an addressee. Haviland observes that gaze can also be withheld to exclude or disallow participation from a potential interlocutor. Similarly, in a study of sign language interactions in a classroom setting in Iquitos, Peru, Goico (2020) describes the use of eye gaze – and the withholding of gaze – to manage turns in conversations between deaf and hearing students who sign with each other regularly at school. In both of these examples, local sign languages are used between skilled deaf and hearing signers and, similar to discussions of turn initiations in national sign languages like ASL, signers typically establish eye gaze with their interlocutor before initiating a turn.

In a comparative study of child sign socialization from three communities, including “Z” as well as signers from the village of San Juan Quiahije, in Oaxaca, Mexico, and the town of Nebaj, in Guatemala, Hou et al. (2021), describe patterns of attention-getting, turn-taking, and physical orientation in conversations between children and adults in local sign languages. In these three sign language communities, gaze serves as a significant regulator for turn-taking. Adult signers establish eye gaze with their interlocutor before they begin signing, and in San Juan Quiahije and Nebaj, adult signers used waves, taps, and knocking on a table surface, prior to beginning their turns. Beyond the use of similar signals to initiate turns, however, Horton et al. find differences in the degree to which adult signers engage child signers directly in conversation. The authors argue that this variation may be influenced by local cultural practices where children tend to learn through observation rather than be explicitly socialized through child-directed language patterns.

While this body of work on emerging sign languages is still developing, we hope that going forward these researchers will go beyond single signer informants and collect video-recordings of caregiver-child dyads and multi-party conversations as well to explore conversational and pragmatic practices in signed languages. In many of these communities, multi-party interactions are more common for children to experience because several families can live together within a compound, or children are cared for by extended family networks or older siblings. It will be especially interesting to note the timing of turns and whether overlaps are more likely to exist in young sign languages.

## Discussion

In this article we have reviewed studies that explore the relationship between language modality and turn-taking, the trajectory of turn-taking skills in infancy and childhood, as well as the development of turn-taking in diverse social ecologies for sign language acquisition. This work sets up several puzzles, as well as areas for future investigation. In terms of the relationship between language modality and turn-taking, by some measures, turn-timing in sign languages closely patterns with that of spoken languages for particular turn types (polar question and answer sequences). Further, conversations in sign and speech seem to be generally guided by the same underlying principle of minimizing overlaps as well as gaps between turns, lending support for a universal “interaction engine” (Levinson, 2019). However, studies that have attempted to measure turn timing highlight the challenge of identifying sign boundaries. It remains somewhat unclear whether sign language conversations have comparatively more overlap of turns or if overlapping turns may last longer, on average, than spoken language turns. We do not know what turn timing looks like for DHH infants and children in interactions with their caregivers. This would provide a useful datapoint to understand the time course of turn-taking development in sign language acquisition.

For the acquisition of turn-taking in childhood, unlike other domains of language use, children seem to have the ability and desire to engage in turn-taking activities and behaviors from a very young age. As they develop, there is some evidence that increasing linguistic and social skills may slow down their prelinguistic alternations with caregivers. Thus, even though some of this ability appears quite early, its time course is actually quite protracted and interacts with other developmental milestones (Casillas et al., 2016).

The early availability of turn-taking behaviors has implications for DHH children acquiring a sign language at older stages of development. Particularly in combination with evidence that some pragmatic cues for turn-taking in sign languages appear to be available to hearing adults with co-speech gesture experience but no sign language experience (de Vos et al., 2022). These two pieces of evidence might suggest that DHH signing children would have intuitions about pragmatics and turn-taking in sign language, even if they enter the signing classroom with minimal sign language experience from home. Hypothetically, they should be able to draw on innate, early abilities and/or cues that are available to all language users. But we do not see this pattern in much of the data from classrooms where children are acquiring sign languages. The DHH children who enter the signing classroom with appropriate turn-taking abilities and pragmatic skills typically have sign exposure early in their home environment. Given the fact that many late learners of sign languages do not appear to have natural instincts for visual attention that will grant them access to signed interactions in the classroom, we review literature that discusses teacher practices.

If the classroom environment is the primary site of sign language socialization for DHH children from hearing families who do not sign, one strategy might be for teachers to emulate DHH signing parents. Based on the literature documenting deaf signing caregivers’ practices, this entails creating an immersive signing environment in which the adult signer waits for the novice signer’s visual attention or adapts their signing to be within the novice signer’s visual field. In the classroom, this might involve waiting for DHH students to notice or develop their visual monitoring skills without explicit prompts or scaffolding. This is not, however, what studies have found is the predominant pattern in signing classrooms. Teachers appear to often use very explicit socializing strategies, though this may vary significantly based on the activity. In a recent study of shared story-time in kindergarten and first grade classrooms, Hou et al. (2021) found that signing teachers were less likely to explicitly direct students’ attention than speaking teachers in oral classrooms with DHH students who were using spoken English. There are a number of significant differences between the home environment for young DHH signers who have DHH signing parents and DHH children from hearing families at school, some of these are summarized in Table 8.

**TABLE 8:** Differences in the social ecologies of home and school as primary sites of sign language acquisition.

	National sign language acquisition in deaf signing families	National sign language acquisition in the classroom	Local sign language acquisition in signing families
Participant framework	Dyadic	Multiparty	Multiparty
Contexts of use	Home (informal)	School (institutional)	Home (informal)
Age of acquisition	Younger (from birth)	Older (school-age)	Variable
Style of interaction	Socializing	Didactic/instructional	Socializing

As discussed across several sections, the social demands and affordances of these three diverse settings for acquisition have significant implications for the development of turn-taking skills. While national sign language acquisition that occurs in deaf families at home may be characterized by less input that is very targeted to the individual child, this may not be feasible in a classroom setting. Further, the DHH child is immersed in sign language and visual-manual turn-taking activities from early in development and in interactions that are primarily about socialization. Before the DHH child is fully mobile and prior to their acquisition of linguistic skills, they can be the recipient of targeted input that is adapted to their attentional abilities. National sign language acquisition in the signing classroom happens for DHH children who are already mobile and who are already part of families that are using speech and auditory cues for turn-taking. Thus, they are getting less sign language input in a context in which there is significantly more competition – both for their visual attention and for the conversational signing floor – as they are typically engaged in multiparty interactions with both their peers and their teacher. We still lack significant information about how turn-taking transpires in multiparty adult signing conversations, but in classrooms, many teachers seem to focus on managing turns so that students do not overlap with one another, and on supporting DHH students who are struggling to figure out where to direct their attention (Singleton and Crume, 2010). The acquisition of local sign languages at home provides an interesting counterpoint to the national sign language examples. Similar to national sign language acquisition at home, the signing in these contexts may not be overly marked for the child, depending on ideologies of language socialization in the signing community. Child signers may need to learn to develop their turn-taking and visual attention skills with minimal explicit instruction or guidance. Similar to national sign language acquisition in the classroom, however, children acquiring local sign languages may typically be observers of multiparty signed conversations, rather than participants.

Deaf or hard of hearing children acquire sign languages in highly variable contexts, making it difficult to isolate the relative contributions of language modality, linguistic and cognitive development, and social setting, to any language practice. By gathering more thorough data from naturalistic interactions across these ecologies, we will be better able to piece together the emergence of turn-taking skills in sign language development, and interrogate the relationship between modality and turn-taking in conversation.

## Author contributions

Both authors listed have made a substantial, direct, and intellectual contribution to the work, and approved it for publication.

## Footnotes

1 We use “deaf” and the acronym DHH to refer to children with a range of hearing thresholds and speaking or signing preferences. In some research on DHH people, it has been common practice to capitalize the word “Deaf” when referring to members of the deaf signing community, this practice has been debated in more recent work (Kusters et al., 2017; Pudans-Smith et al., 2019). In cases where we discuss studies in which the authors make the d/Deaf distinction in their work, we maintain their usage of “Deaf.”

2 See Hou and de Vos (2022) for a recent discussion of terminological distinctions in sign language research.

3 We thank a reviewer for pointing out that cues to turn-taking have also been studied for tactile sign (Mesch, 2002). A recent study also explores turn-taking in conversations with deaf-blind signers of Bay Islands Sign Language Ali et al. (2021).

4 Here we refer to DHH children in hearing/speaking families. Although these children may have limited or reduced access to the linguistic information in the spoken language around them, they will still be able to access some of this input, as well as the co-speech gestures that speakers produce (see Koulidobrova and Chen-Pichler, 2021, for a discussion of diverse early language experiences of DHH children in hearing/speaking contexts).

5 Estimates suggest that approximately 5% of DHH children in the United States are born into a family with one or more DHH parents (Mitchell and Karchmer, 2004).

6 For spoken language development of DHH children at home: Smolen et al. (2021); Arora et al. (2020), Lederberg and Everhart (2000). For spoken language development of DHH children in oral classrooms: Duncan and Lederberg (2018); Lloyd et al. (2001), Vandell and George (1981); Wood et al. (1982).

7 The mothers and children were deaf and used British Sign Language, BSL (Harris et al., 1989, p. 84).

8 Three ASL parent–child dyads in the United States and three LSE parent–child dyads in Spain (Holzrichter, 2000).

9 This is only true for DHH children who are enrolled in schools that use sign language as the mode of instruction. Many DHH children are mainstreamed or enrolled in schools where the primary language of instruction is spoken language.
